# Synthesis, Characterization, and Biological Activity of N^1^-Methyl-2-(1H-1,2,3-Benzotriazol-1-y1)-3-Oxobutan- ethioamide Complexes with Some Divalent Metal (II) Ions

**DOI:** 10.1155/2008/479897

**Published:** 2008-02-28

**Authors:** Nouria A. Al-Awadi, Nadia M. Shuaib, Alaa Abbas, Ahmed A. El-Sherif, Ali El-Dissouky, Esmaeil Al-Saleh

**Affiliations:** ^1^Chemistry Department, Faculty of Science, Kuwait University, P.O. Box 5969, Safat 13060, Kuwait; ^2^Microbiology Program, Department of Biological Sciences, Faculty of Science, Kuwait University, P.O. Box 5969, Safat 13060, Kuwait

## Abstract

A new series of Zn^2+^, Cu^2+^, Ni^2+^, and Co^2+^ complexes of N^1^-methyl-2-(1H-1,2,3-benzotriazol-1-yl)-3-oxobutanethioamide (MBOBT), HL, has been synthesized and characterized by different spectral and magnetic measurements and elemental analysis. IR spectral data indicates that (MBOBT) exists only in the thione form in the solid state while 13C NMR spectrum indicates its existence in thione and thiole tautomeric forms. The IR spectra of all complexes indicate that (MBOBT) acts as a monobasic bidentate ligand coordinating to the metal(II) ions via the keto-oxygen and thiolato-sulphur atoms. The electronic spectral studies showed that (MBOBT) bonded to all metal ions through sulphur and nitrogen atoms based on the positions and intensity of their charge transfer bands. Furthermore, the spectra reflect four coordinate tetrahedral zinc(II), tetragonally distorted copper(II), square planar nickel(II), and cobalt(II) complexes. Thermal decomposition study of the complexes was monitored by TG and DTG analyses under N_2_ atmosphere. The decomposition course and steps were analyzed and the activation parameters of the nonisothermal decomposition are determined. The isolated metal chelates have been screened for their antimicrobial activities and the findings have been reported and discussed in relation to their structures.

## 1. INTRODUCTION

Compounds containing triazoles have attracted much interest because of their biological applications [1–4]. Furthermore, triazoles appear frequently in the structures of various natural products [5]. Triazole containing compounds appear in many metabolic products of fungi and primitive marine animals. Many triazoles having different functionalities are used as dyes and as photographic chemicals [6]. The coordination chemistry of triazole and benzotriazole derivatives was studied due to their importance in industry, agriculture and their biological activity. The mercapto group often coordinated to metal ions in many biological molecules [7] and information about the relative reactivity of the coordinated mercapto group might give insight into the specific reactivity of active sites in some metalloproteins. On the other hand, some of the transition metals present in trace quantities are essential elements for biological systems. In view of the above facts and in continuation of our interest in studying the ligating behavior of such compounds [8–11], we aim to (i) synthesize and characterize the solid complexes of the newly ligand containing both the triazole and thioamide moieties, *N^1^*-methyl-2-(1*H*-1,2,3-benzotriazol-1-yl)-3-oxobutanethioamide (MBOBT), HL, **I** with Zn^2+^, Cu^2+^, Ni^2+^, and Co^2+^, (ii) study their thermal decomposition characteristics and determine the different thermodynamic parameters, and (iii) investigate their antimicrobial effects towards some Gram-positive and Gram-negative bacteria.

## 2. EXPERIMENTAL

### 2.1. Materials and reagents

All chemicals were reagent grade quality obtained from BDH and Aldrich Chemical Companies and used as received.

### 2.2. Synthesis of MBOBT

The organic ligand was prepared according to the previously reported method [12].

### 2.3. Synthesis of metal complexes

The complexes are synthesized by the general method, namely, a solution of hydrated metal(II) acetate(0.001 mol; 0.22, 0.19, 0.25, and 0.25 g of Zn (OAc)_2_ ⋅ 2H_2_O, Cu(OAc)_2_ ⋅ H_2_O, Ni(OAc)_2_ ⋅ 4H_2_O, Co(OAc)_2_ ⋅ 4H_2_O, resp.) in EtOH (30 mL), Cu(OAc)2.H_2_O was dissolved in M_e_OH, and (0.0022 mol, 0.53 g) in EtOH (25 mL) followed by the addition of 3–5 mL triethylamine (TEA). The reaction mixture was refluxed for 2-3 hours on a water bath and then cooled to the room temperature. The solid product in each case was filtered off, washed several times with EtOH, Et_2_O, and dried in vacuum over P_4_O_10_.

### 2.4. Screening for antibacterial activity

The synthesized MBOBT and its four metal(II) complexes were screened in vitro for their antibacterial activity against five Gram-positive (*Staphylococcus aureus*,*Staphylococcus hominis*, *Bacillus sp^1^*, *Bacillus sp^2^*, *and* *Bacillus sp^3^*) and three Gram-negative (*Escherichia coli*, *Salmonella sp^1^* *and* *Salmonella sp^2^*) bacterial strains using gel diffusion and respirometric method. The gel diffusion method was used as previously described [13]. Bacterial cultures were grown overnight on nutrient agar (NA) plates. Bacterial biomass was suspended in 0.9% saline and adjusted to an optical density (OD) of 0.02 at *λ* 600 nm. Bacterial suspensions were spread on the NA plates using sterile cotton swaps. Uniform wells were created in the NA plates using a cork-borer (6 mm). Synthesized chemicals (dissolved in ethanol) were transferred (100 *μ*L, 0.1 mg) into the wells and ethanol was used as control. Plates were incubated for 24 hours at 30°C and the diameter of inhibition zones around the wells was measured in centimeter (cm). Each test was conducted in triplicate and the mean with standard deviation was calculated. The inhibitory effects of synthesized MBOBT and its four metal(II) complexes in ethanol as solvent on bacterial respiration were also investigated using the method of Al-Saleh and Obuekwe [14]. Synthesized chemicals (0.5 and 1 mg) were transferred to sterile bottles containing 49 mL nutrient broth and bacterial culture (1 mL of overnight culture, OD 1 at *λ* 600 nm). Bottles were connected to respirometer (Micro-Oxymax Columbus Instruments) and incubated in a shaking water bath at 30°C. Bottles with sterile nutrient broth were used as control. Experiments were conducted in triplicates and the amount of carbon dioxide evolved was plotted against time. In order to clarify any participating role of EtOH in the biological screening, separate studies were carried out with the solutions without the complexes and they showed less or no activity against any bacteria.

**Physical measurements and analysis.**

CHNS analysis was obtained using LECO-CHNS 932 Analyzer. FT-IR spectra were recorded as KBr discs with Schimadzu 2000 FT-IR spectrophotometer. Electronic spectra were accomplished by Carry Varian 5 UV/Vis spectrophotometer. The room temperature magnetic susceptibility measurements for the complexes were determined by the Gouy balance using Hg[Co(NCS)_4_] as a calibrant. Thermal analysis measurement was performed by using a dynamic nitrogen atmosphere with a TGA-50 Shimadzu thermogravimetric analyzer at a flow rate of 50 mL⋅min^−1^. The heating rate was 10°C⋅min^−1^ and the sample sizes ranged in mass from 6 to 8 mg. ^1^H NMR was determined on a Bruker DPX 400 MHz superconducting spectrometer in CDCl_3_ and DMSO-d_6_ as solvents and using TMS as internal standard.

## 3. RESULTS AND DISCUSSION

### 3.1. General

The reaction of (MBOBT) with metal ions under stirring and different mole ratios gave the complexes presented in Table 1 and their formulation is based on the obtained elemental analyses. The complexes are air stable, insoluble in the most organic solvents and water but freely soluble in DMF and DMSO. The complexes have higher melting points than their corresponding ligands indicating that they are thermally stable. This could be attributed to the formation of chelate rings and/or increased in conjugation due to complexation.

### 3.2. Characterization of the MBOBT and its solid complexes

#### 3.2.1. NMR and IR spectra of MBOBT and its complexes

The 13C NMR spectrum of *MBOBT* in d_6_-DMSO was recorded. Despite expecting signals for only 10 carbons, twenty carbon signals appeared with the spectra indicating that at least in DMSO, the molecule exists as an equilibrium mixture of two forms (I A and I B). The existence of a signal at *δ* 76.16 ppm characteristic of an sp^3^ carbon indicated clearly that one of these two forms is A. In the C=O region two carbonyl carbons at *δ* 197.9 and 192.3 ppm are detected indicating the presence of C=O in both forms. The spectrum exhibits only one C=S signal at *δ* 197.98 ppm (should be appeared at *δ* 173 ppm). Furthermore, the spectrum displays a signal at *δ* 173.4 ppm characteristic of an sp^2^ carbon in accordance with the assumption of the second form B.

The infrared spectra of MBOBT and its different complexes are recorded as KBr discs and main bands with their tentative assignments given in Table 2. The spectrum of MBOBT does not show *ν*(SH) band at 2600–2500 cm^−1^, in which this stretching frequency is generally expected and is therefore mainly in the thioketo form [15]. The spectrum of MBOBT displays four bands 1499, 1375, 1073, and 836 cm^−1^ assigned to the thioamide bands, namely I, II, III, and IV, contains a thioamide group (HNC=S), and has contribution from *δ*(C–H) + *δ*(N–H), *ν*(C=S) + *ν*(C=N) + *ν*(C–H), *ν*(C–N) + *ν*(C–S), and *ν*($\text{C}\dddot{-}\text{S}$ ), respectively [16, 17]. These thioamide bands III and IV are strongly shifted to lower wavenumbers in the spectra of all complexes supporting sulphur donation and deprotonation of the ligand as well. Furthermore, the thioamide bands I and II are not greatly affected by complexation suggesting the nonbonding nature of the nitrogen to the metal ion. The spectrum of MBOBT displays only a weak C=O absorption at 1644 cm^−1^ and medium-strong band at 3280 cm^−1^ due to *υ*(NH). Shifting position and decreasing intensity of the *υ*(C=O) and shifting of the *υ*(NH) to longer wavelength and increasing intensity may be due to hydrogen bonding formation between these two groups. As expected, CH stretches for C–H linked to sp^2^ and sp^3^ carbon appeared at 3045 and 2947 cm^−1^, respectively.

All these data suggest the presence of the free MBOBT in the form A in the solid state. This band is red shifted by ca. 18–44 cm^−1^ upon complex formation supporting the bonding of oxygen to the metal ion. Accordingly, BMMB acts as a monobasic bidentate ligand coordinated to the metal ions through the deprotonated thiolo-sulphur and keto-oxygen atoms [18, 19].

#### 3.2.2. Electronic spectra and magnetic studies

The electronic spectra of the complexes, Table 3, show intense bands at 26700–28600 and 29300–29700 cm^−1^ attributable to the intra-ligand O–M(II) transitions suggesting the bonding of the ligand oxygen to the metal ion. The spectra also exhibit a strong band at 22300–24700 cm^−1^ characteristic of S–M(II) LMCT transition and further support the bonding of the ligand to the metal ion via a sulphur atom.

The electronic spectrum of [L_2_Zn]⋅H_2_O shows intense bands at 29280, 26850, and 23700 cm^−1^ which are assigned to the intraligands O → Zn(II) and S → Zn(II) LMCT, respectively. These spectral features indicate the bonding of BMMB to the Zn(II) via oxygen and sulphur atoms. The spectrum shows no bands in the region below 23000 cm^−1^ which is in accordance with the d^10^ electronic configuration of Zn(II).

Copper(II) complex [L_2_Cu] gives a room temperature magnetic moment value of 1.78 B.M. characteristic of magnetically diluted copper(II) species. Its electronic spectrum displays only an intense band at 26200 cm^−1^ which is attributed to the intraligand (ligand localized) and LMCT transitions and characteristic of a tetragonally distorted copper(II) complexes.

The electronic spectrum of [L_2_Ni] displays bands at, 16890, 19950, and 24200 cm^−1^ assignable to ^1^A_1g*υ*_ → ^1^A_2g_, (*υ*_1_), ^1^A_1g_ → ^1^B_1g_(*υ*_2_), and ^ 1^A_1g_ → ^1^E_g_(*υ*_3_) transitions, respectively, characteristic of square planar nickel(II) complexes. The first two bands are pure d-d transitions while the *υ*_3_ band obviously enveloped by a strong CT transition. The assumed square planar geometry for this complex is confirmed from the value of its room temperature magnetic moment of zero.

The room temperature magnetic moment of [L_2_Co] of 2.70 B.M. is more than that of low spin octahedral and lower than the values characteristic of tetrahedral cobalt(II) complexes. Furthermore, these values are similar to that reported for the square planar cobalt(II) complexes [20, 21]. The electronic spectrum of the complex exhibits two bands at 8500 and 19960 cm^−1^ characteristic of square planar cobalt(II) complexes with a transition involving nonbonding rather antibonding orbitals. In a strong field, the ground state is probably ^ 2^A_1g_ with the configuration of e_*g*_^4^b_2*g*_^2^a_1*g*_^1^.

#### 3.2.3. Thermal analysis

**Figure 1:**
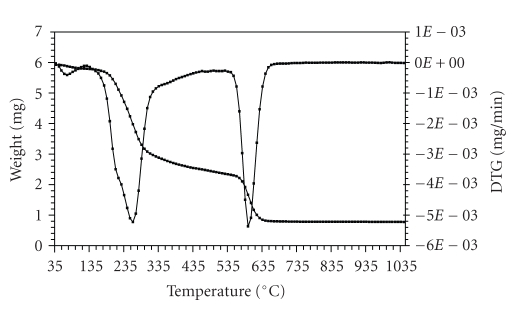
TG and DTG plots of [L_2_Zn]⋅H_2_O.

**Figure 2:**
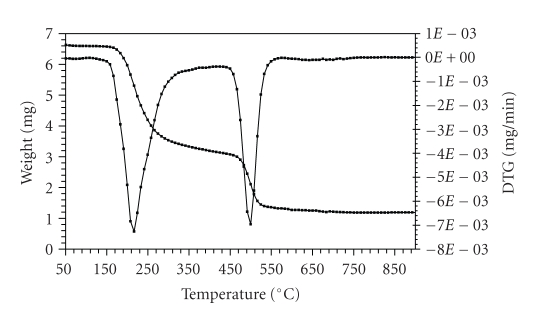
TG and DTG plots of [L_2_Cu].

**Figure 3:**
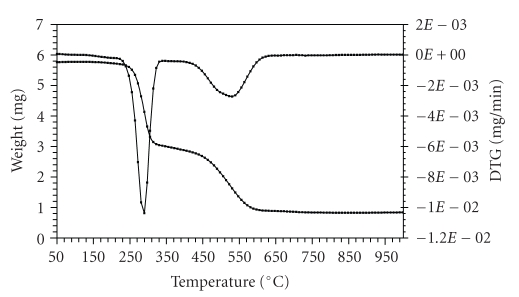
TG and DTG plots of [L_2_Ni].

**Figure 4:**
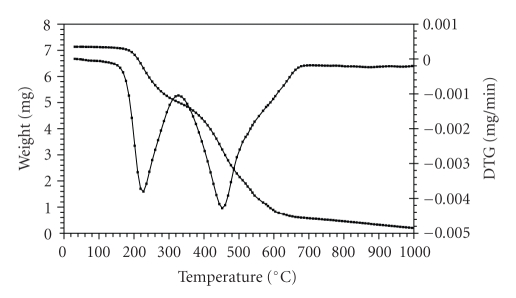
TG and DTG plots of [L_2_Co].

The thermogravimetric (TG) and the derivative thermogravimetric (DTG) plots of the complexes in the 25–1200°C range under N_2 _ are shown in Figures 1–4. Their stepwise thermal degradation data are given in Table 4. All complexes show two-stage mass loss except [L_2_Zn]⋅H_2_O shows three decomposition steps.

The TG and DTG curves of [L_2_Zn]⋅ H_2_O are shown in Figure 1. The TGA curve of this complex shows three stages of decomposition within the temperature range (32–654°C). The first step of decomposition within the temperature range (32–120°C) corresponds to the loss of water molecule of hydration with mass loss of 2.9% (calcd. 3.1%). The second step (136–328°C) corresponds to the loss of two benzotriazole (BTA) moities and two acetylene molecules (mass loss 49.2%; calcd. 49.8%). The third step (545–654°C) corresponds to the loss of SO_2 _ and L_1_ molecules (mass loss 30.1%; calcd. 29.8%). The energies of activation were 43.73, 37.1 and 24.4 kJ mol^−1^ for the first, second, and third steps, respectively. The total mass loss up to 654°C is in agreement with the formation of ZnS as the final residue (TG 16.1%, calcd. 16.4%).

The thermogram given in Figure 2 of [L_2_Cu] exhibits two significant thermal events within the temperature range (158–549°C). The first step of decomposition within the temperature range (158–316) corresponds to the loss of two BTA and two acetylene molecules with a mass loss 51.2% (calcd. 51.6%). The second step (449–549°C) corresponds to the loss of SO_2_ and L_1_ molecules (mass loss 30.9%; calcd. 31.2%). The energies of activation were 73.83 and 26.36 kJ mol^−1^ for the first and second steps, respectively. The total mass loss up to 549°C is in agreement with the formation of CuS as the final residue (TG 17.2%, calcd. 17.9%).

The TG and DTG curves of [L_2_Ni] are shown in Figure 3. The TGA curve shows two stages of decomposition within the temperature range (222–608°C). The first step of decomposition within the temperature range (222–331°C) corresponds to the loss of two BTA and two acetylene molecules with a mass loss of 52.1% (calcd. 51.8%). The second step (414–608°C) corresponds to the loss of L_1 _ molecule (mass loss 31.4%; calcd. 31.1%). The energies of activation were 64.3 and 16.7 kJ mol^−1^ for the first and second steps, respectively. The total mass loss up to 608°C is in agreement with the formation of NiS as the final residue (TG 16.4%, calcd. 17.1%).

The [L_2_Co] complex is thermally stable up to 670°C; see Figure 4. From the TG curve it appears that the complex decomposes in two stages over the temperature range 160–670°C. The first decomposition occurs between 160–237°C with mass loss of (calcd. 51.6%) and the second decomposition starts at 237°C and ends at 670°C with a 31.1% mass loss (calcd. 31.4%). The first step of decomposition corresponds to the loss of two BTA and two acetylene molecules while the second step corresponds to the loss of SO_2_ and L_1_ molecules. The energies of activation were 59.5 and 14.0 kJ mol^−1^ for the first and second steps, respectively.

#### 3.2.4. Kinetic data for the decomposition of complexes

The thermodynamic parameters of decomposition processes of complexes, namely, activation energy (*E_a_*), enthalpy (ΔH*), entropy (ΔS*), and Gibbs free energy change of (ΔG*) were evaluated graphically by employing the Coats-Redfern method [22, 23]. This method, reviewed by Johnson and Gallagher [23] as an integral method assuming various orders of reaction and comparing the linearity in each case to select the correct order by using

(1) $$\begin{array}{ll} \text{log}\left[\frac{1-{\left(1-\alpha \right)}^{1-n}}{T^{2}(1-n)}\right] & =\text{log}\left[\frac{\mathrm{AR}}{\theta E_{a}}\left(1-\frac{2\mathrm{RT}}{E_{a}}\right)\right]\\ & \;-\frac{E_{a}}{2.303\mathrm{RT}}\;\text{for}\;\;n\neq 1,\\ \text{log}\left\{\frac{-\text{log}\;(1-\alpha )}{T^{2}}\right\} & =\text{log}\left[\frac{\mathrm{AR}}{\theta E_{a}}\left(1-\frac{2\mathrm{RT}}{E_{a}}\right)\right]\\ & \;-\frac{E_{a}}{2.303\mathrm{RT}}\;\text{for}\;\;n=1, \end{array}$$

where *α* is the fraction of sample decomposed at time *t*, *T* is the derivative peak temperature, *A* is the frequency factor, *E_a_* is the activation energy, *R* is the gas constant, *θ* is the heating rate, and (1 − (2*RT*/*E_a_*)) ≅ 1. A plot of log{− log(1 − *a*)/*T* ^2^} versus 1/*T* gives a slope from which the *E_a_* was calculated and A (Arrhenius factor) was determined from the intercept. Trials of these plots were made by assuming the orders 0, 1/2, and 1 and the best plot was obtained for the first order. The entropy of activation was calculated using [24]

(2) $$\Delta S^{*}=2.303R\left[\text{log}\;\left(\frac{\mathrm{Ah}}{\mathrm{kT}}\right)\right],$$

where *h* and *k* stand for the Planck and Boltzmann constants, respectively, and *T* is the peak temperature from the DTG curve. The free energy of activation ΔG* and the enthalpy of activation ΔH* are calculated using (3),

(3) $$\begin{array}{l} \Delta H^{*}=E_{a}-\mathrm{RT},\\ {\Delta G}^{*}={\Delta H}^{*}-{T\Delta S}^{*}. \end{array}$$

The kinetic data obtained from the nonisothermal decomposition of the complexes are given in Table 5.

The activation energy of the complexes is expected to increase with decreasing metal ion radius [25, 26]. The smaller size of metal ions permits a closer approach of the ligand.

Hence, the ΔE* value in the first stages for the Cu(II) complex is higher than those of Ni(II), Co(II), and Zn(II) complexes [27–29]. The calculated ΔE* values using Coats-Redfern method for the first-stage decomposition of the complexes are found to be

Δ*E* ^*^_Cu_ = 73.8 KJ mol^−1^ > Δ*E* ^*^_Ni_ = 64.3 KJ mol^−1^ > Δ*E* ^*^_Co_ = 59.5 KJ mol^−1^ which is in accordance with *r*_Cu(II)_ **=** 70 pm < *r*_Ni_(II) = 72 pm < *r*_Co(_II) = 74 pm.

The same decomposition kinetics is also true for the ΔE* values of the second stage decomposition which was found to be in the following order:

Δ*E* ^*^_Cu_ = 26.3 KJ mol^−1^ > Δ*E* ^*^_Ni_ = 16.7 KJ mol^−1^ > Δ*E* ^*^_Co_ = 14.0 KJ mol^−1^

The negative values of ΔS*, see Table 5, indicate that the reaction rates are slower than normal [30] which is consistent with the results reported previously [31]. Furthermore, these data indicate that the activated complexes have more ordered structure than the reactants [29–31].

#### 3.2.5. Biological activity

**Figure 5:**
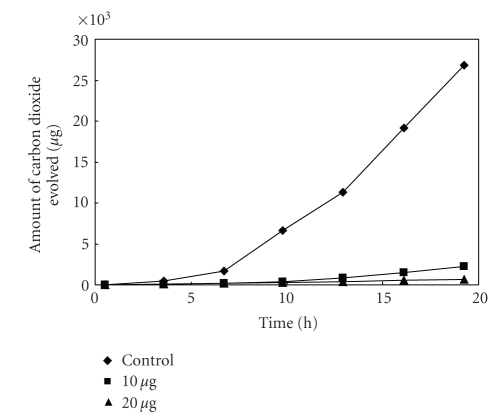
Effect of [L_2_Cu] on the respiration of *Bacillus* sp^1^.

**Figure 6:**
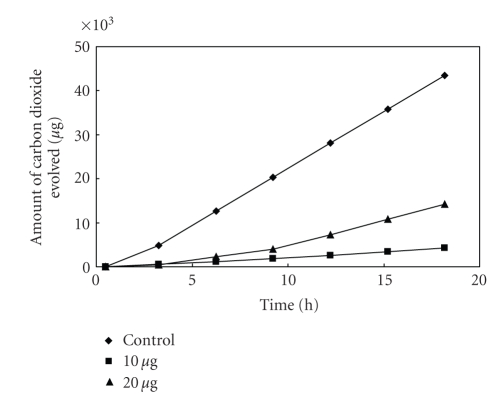
Effect of [L_2_Cu] on the respiration of *Bacillus* sp^2^.

**Figure 7:**
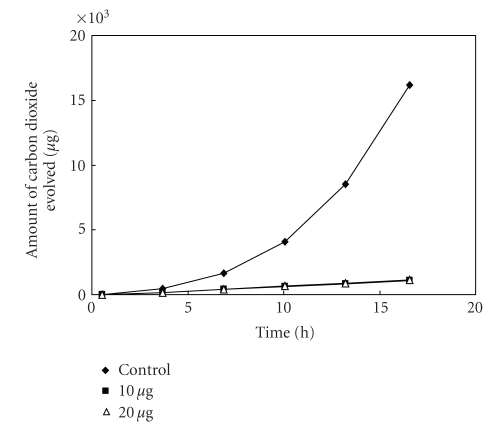
Effect of [L_2_Cu] on the respiration of *Bacillus* sp^3^.

**Figure 8:**
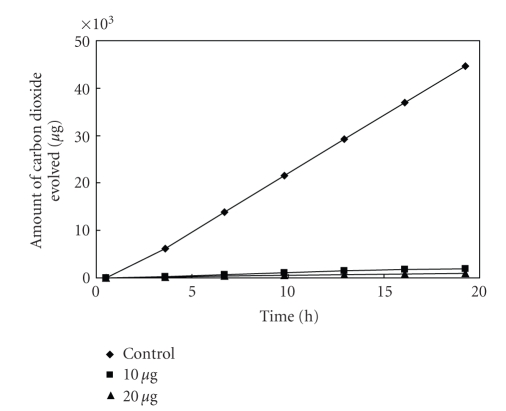
Effect of [L_2_Cu] on the respiration of *E. coli*.

**Figure 9:**
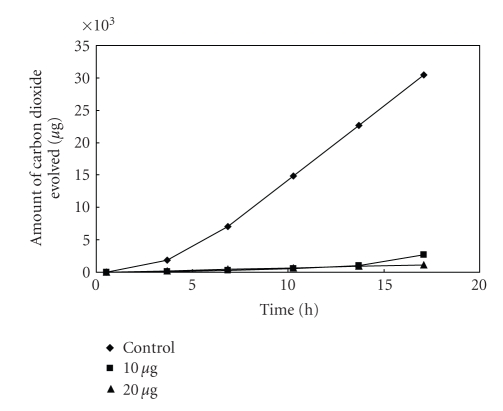
Effect of [L_2_Cu] on the respiration of *Salmonella* sp^1^.

**Figure 10:**
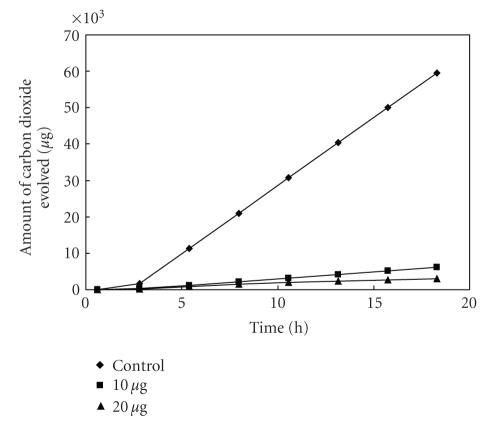
Effect of [L_2_Cu] on the respiration of *Staphylococcus aurous*.

**Figure 11:**
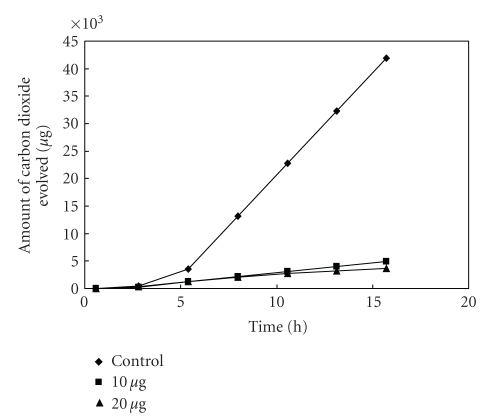
Effect of [L_2_Cu] on the respiration of *Staphylococcus hominis*.

**Figure 12:**
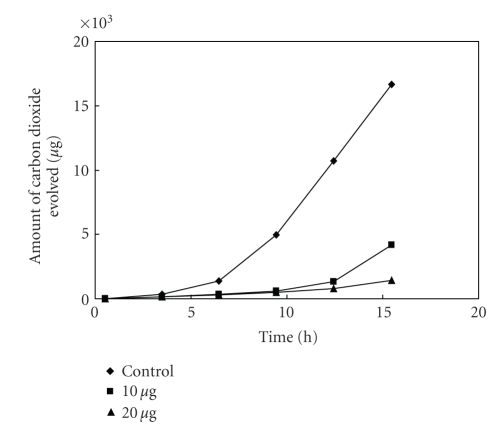
Effect of [L_2_Zn]⋅H_2_O on the respiration of *Salmonella* sp^1^.

**Figure 13:**
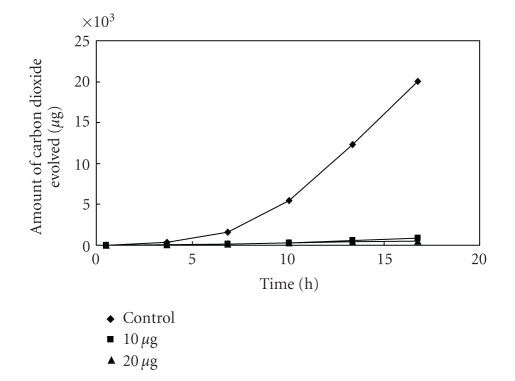
Effect of [L_2_Zn]⋅H_2_O on the respiration of *Bacillus* sp^1^.

**Figure 14:**
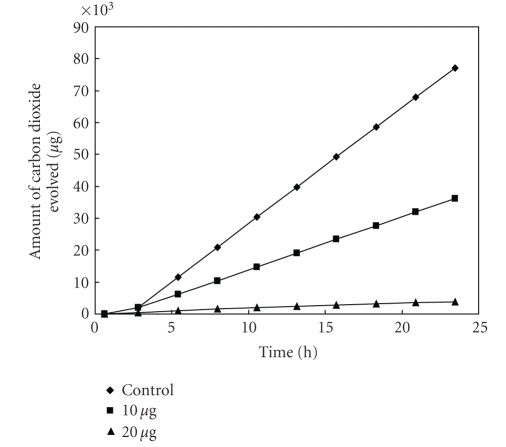
Effect of [L_2_Zn]⋅H_2_O on the respiration of *Bacillus* sp^2^.

**Figure 15:**
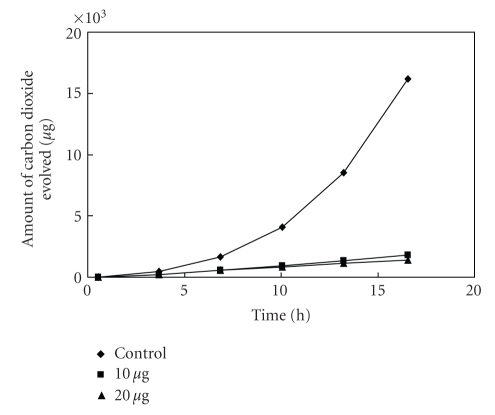
Effect of [L_2_Zn]⋅H_2_O on the respiration of *Bacillus* sp^3^.

The antibacterial activity of MBOBT and its metal(II) complexes are given in Tables 6 and 7 and the average of three experimental data for [L_2_Zn]⋅H_2_O and [L_2_Cu] are shown in Figures 5–15. The results show that (i) the complexes exhibit inhibitory effects towards the activity of gram-positive and gram-negative bacteria in contrast to the parent organic ligand which is biologically inactive under the experimental conditions, (ii) all complexes are inactive towards *Salmonella* sp^2^ and only [L_2_Cu] is active towards *Staphylococcus aurous,* and (iii) copper(II) complex has a wide spectrum with respect to the studied bacteria. As previously reported, the metal salts do not exhibit antimicrobial activity [32–36]. The biological activity of the metal complexes is governed by the following factors [36]: (i) the chelate effect of the ligands, (ii) the nature of the donor atoms, (iii) the total charge on the complex ion, (iv) the nature of the metal ion, (v) the nature of the counter ions that neutralize the complex, and (vi) the geometrical structure of the complex [35]. Furthermore, chelation reduces the polarity of the metal ion because of partial sharing of its positive charge with the donor groups and possibly the *π*-electron delocalization within the whole chelate ring system that is formed during coordination [33]. These factors increase the lipophilic nature of the central metal atom and hence increasing the hydrophobic character and liposolubility of the molecule favoring its permeation through the lipid bilayer of the bacterial membrane. This enhances the rate of uptake/entrance and thus the antibacterial activity of the testing compounds. Accordingly, the antimicrobial activity of the four complexes can be referred to the increase of their lipophilic character which in turn deactivates enzymes responsible for respiratory processes and probably other cellular enzymes, which play a vital role in various metabolic pathways of the tested bacteria. Also it is proposed that the action of the toxicant is the denaturation of one or more proteins of the cell and this impairs normal cellular process. According to the data given in Tables 6 and 7, the antimicrobial activity can be ordered as [L_2_Cu] > [L_2_Zn]⋅H_2_O > [L_2_Ni] > [L_2_Co], suggesting that the lipophilic behavior increases in the same order. Since all complexes (i) have the same donating atoms which are S/O with the same coordination number (C.N. for each is 4), (ii) have the same chelate effect (all form two 6-membered chelating rings), (iii) are neutral and there are no counter ions, and (iv) have the same oxidation number in their complexes (M^2+^), therefore, the more effective factors are the geometrical shape and the nature of the central atoms. According to the spectral and magnetic studies, (i) copper has a tetragonal distortion (distorted to a tetrahedral geometry); (ii) cobalt and nickel have a square planar; (iii) zinc is associated with a tetrahedral geometry. Therefore, the higher antimicrobial activity can be referred to their similar structure which is the tetrahedral. This structure increases the lipophilicity of the central atom by decreasing the effective nuclear charge (polarity) of the Zn(II) and Cu(II) more than the square planar structure of Co(II) and Ni(II). The higher antimicrobial activity of copper(II) complex relative to the zinc(II) complex may be referred to the presence of water molecule in the formula of the later complex, also copper(II) may form stronger copper(II)-ligand bond than Zn(II)-ligand bond and this in turn increases the lipophilic character of copper(II) complex than zinc(II) complex. The redox activity of copper compared to zinc, which is redox neutral, may be taken as an additional reason for the higher activity of copper relative to zinc complex.

## 4. SUMMARY AND CONCLUSIONS

The interaction of the newly synthesized MBOBT with Zn^2+^, Cu^2+^, Ni^2+^, and Co^2+^ leads to the formation of neutral complexes [L_2_M]⋅nH_2_O. Their structures and formation are determined using microanalysis, magnetic, and different spectral tools. Copper and Zn(II) complexes are of a distorted tetrahedral whereas cobalt(II) and nickel(II) complexes are associated with square planar(II) structures. The thermal analysis data showed that the stability of the complexes can be ordered as [L_2_Cu] > [L_2_Ni] > [L_2_Co] > [L_2_Zn]⋅H_2_O. Furthermore, the negative values of ΔS* indicate that the reaction rates are slower than normal and the activated complexes have more ordered structure than the reactants. The antimicrobial tests showed that (i) the complexes are antimicrobial active while the free ligand BMMB is not, and (ii) the copper(II) complex can be considered as the most promising potent broad spectrum antimicrobial compound among the four complexes, where it is found to be superior to all other complexes against all the test organisms except *Salmonella* sp^2^.

## Figures and Tables

**Scheme 1:**
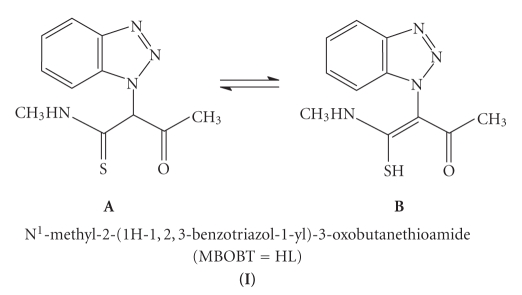


**Table 1:** Elemental analysis [% found (% calculated)], color, and the room-temperature effective magnetic moments (B.M.) of MBOBT and its metal(II) complexes.

Compound	Color	*μ*_eff_	C(%)	H(%)	N(%)	S(%)
HL, C_11_H_12_N_4_SO	Buff	—	53.0 (53.2)	4.7 (4.8)	22.3 (22.6)	12.6 (12.9)
[L_2_Zn]⋅H_2_O, C_22_H_24_N_8_S_2_O_3_Zn	Buff	Diamag	45.3 (45.6)	4.0 (4.2)	19.3 (19.4)	11.0 (11.1)
[L_2_Cu], C_22_H_22_N_8_S_2_O_2_Cu	Light blue	1.82	47.1 (47.4)	4.2 (4.0)	20.1 (20.0)	11.1 (11.4)
[L_2_Ni], C_22_H_22_N_8_S_2_O_2_Ni	Dark blue	Diamag	47.3 (47.6)	4.1 (4.0)	19.9 (20.2)	11.3 (11.5)
[L_2_Co], C_22_H_22_N_8_S_2_O_2_Co	Dark red	2.70	47.7 (47.8)	4.2 (4.0)	19.8 (20.2)	11.7 (11.6)

**Table 2:** Main IR (*ύ*, cm^−1^) bands for MBOBT and its metal(II) complexes. (*w* = week, *m* = medium, *s* = strong.)

Compound	*υ*(OH)	*υ*(NH)	*υ*(C=O)	*υ*(CH_3_)	*υ*(thioamide)			
					I	II	III	IV
HL, C_11_H_12_N_4_SO	—	3280*s*	1644*w*	294*w*7	1499*vs*	1375*m*	1073*m*	836*s*
				3045*w*				
[L_2_Zn]⋅H_2_O, C_22_H_24_N_8_S_2_O_3_Zn	3433*m*	3230*s*	1600*w*	2996*w*	149*m*7	1383*m*	1046*m*	720*m*
				3073*w*				
[L_2_Cu], C_22_H_22_N_8_S_2_O_2_Cu	—	3228*s*	1614*w*	292*w*7	1499*s*	1388*s*	1050*m*	734*m*
				3084*w*				
[L_2_Ni], C_22_H_22_N_8_S_2_O_2_Ni	—	3328*s*	1613*w*	2929*w*	1501*m*	1391*s*	1042*m*	723*m*
[L_2_Co], C_22_H_22_N_8_S_2_O_2_Co	—	3230*s*	1616*w*	2992*w*	1496*m*	1379*s*	1039*w*	718*m*
				3072*w*				

**Table 3:** Electronic spectral data (cm^−1^) for MBOBT complexes.

Compound	Intraligand and CT transitions	d-d transitions
[L_2_Zn]⋅H_2_O, C_22_H_24_N_8_S_2_O_3_Zn	29280, 26850, 23700	—
[L_2_Cu], C_22_H_22_N_8_S_2_O_2_Cu	26200	—
[L_2_Ni], C_22_H_22_N_8_S_2_O_2_Ni	29700, 28600, 24500	24200,19950, 16890
[L_2_Co], C_22_H_22_N_8_S_2_O_2_Co	29620, 26700, 24700	8500, 19960

**Table 4:** Stepwise thermal degradation data obtained from TGA curves for the metal complexes.

Complex	Molar mass	TG range (°C)	DTG_max_ (°C)	Weight loss		Predicated intermediates and final products	Metallic residue (calcd. %) found
				Calcd.	Found		
[L_2_Zn]⋅H_2_O, C_22_H_24_N_8_S_2_O_3_Zn	577.3	32–120	72	3.1	2.9	H_2_O	ZnS
		136–328	261	49.8	49.2	2-BTA	(16.5) 17.4
[L_2_Cu], C_22_H_22_N_8_S_2_O_2_Cu	557.5	158–316	216	51.6	51.2	2-BTA	CuS
		449–549	500	31.2	30.9	SO_2_ + L_1_	(17.2) 17.9
[L_2_Ni], C_22_H_22_N_8_S_2_O_2_Ni	552.7	32–99	78	1.6	1.5	1/2 H_2_O	
		222–331	290	52.1	51.8	2-BTA	NiS
		414–608	531	31.4	31.1	SO_2_ + L_1_	(16.4) 17.1
[L_2_Co], C_22_H_22_N_8_S_2_O_2_Co	552.9	222–331	290	52.08	52.7	2-BTA	CoS
		414–608	531	31.4	29.9	SO_2_ + L_1_	(16.5) 17.4
		222–331	290	52.08	52.7	2-BTA	CoS

BTA = benzotriazole ring, L_1 _= C_6_H_10_N_2_.

**Table 5:** The kinetic parameters for the nonisothermal decomposition of the complexes.

Complex/range (°C)	*T*(a)	*E_a_* (KJ mol^−1^)	*A* (S^−1^)	ΔH* (kJ mol^−1^)	ΔS* (JK^−1^mol^−1^)	ΔG* (kJ mol^−1^)
[L_2_Cu]
90–134	216	73.83	1.5*E*7 + 04	69.76	−169.14	152.47
220–260	500	26.36	4.0*E* − 03	19.93	−299.09	251.13
[L_2_Ni]
222–339	290	64.35	3.*E*7 + 02	69.70	−201.45	173.08
430–608	531	16.71	1.5*E* − 03	10.03	−307.60	257.30
[L_2_Co]
160–327	227	59.53	2.29*E* + 02	55.37	−204.40	157.60
327–670	452	14.04	2.6*E* − 7 04	8.01	−321.10	240.80
[L_2_Zn]⋅H_2_O
32–120	72	43.73	1.91*E* + 03	40.87	−183.70	104.06
153–336	261	37.08	4.15*E* − 01	32.64	−257.50	170.12
544–662	595	24.40	1.29*E* − 03	17.23	−309.50	285.87

^(a)^The peak temperature from the DTG curve.

**Table 6:** Effect of MBOBT and its complexes on the respiration of bacteria. (Results represent percent inhibition of bacterial respiration caused by the addition of 0.5 and 1.0 mg of the test compound. Results are the mean of three independent analyses with standard deviations.)

Compound		Bacteria							
		*Staphylococcus aureus*	*Staphylococcus hominis*	*Bacillus* sp^1^	*Bacillus* sp^2^	*Bacillus* sp^3^	*Escherichia coli*	*Salmonella* sp^1^	*Salmonella* sp^2^
	Amount (mg)								
[L_2_Zn]⋅H_2_O									
	0.5	nil	nil	94.7 ± 6.3	53.2 ± 3.5	84.3 ± 5.6	nil	40.7 ± 2.5	nil
	1.0	nil	nil	93.1 ± 6.3	94.5 ± 6.3	86.1 ± 5.7	nil	79.1 ± 5.3	nil
[L_2_Cu]	0.5	89.6 ± 6.0	87.3 ± 5.8	84.6 ± 5.6	90.3 ± 6.0	88.5 ± 5.8	92.6 ± 6.2	67.7 ± 4.5	nil
	1.0	94.5 ± 6.3	91.0 ± 6.0	95.1 ± 6.5	66.7 ± 4.4	87.9 ± 6.0	93.7 ± 6.4	74.8 ± 5.0	nil
[L_2_Ni]	0.5	nil	nil	68.3 ± 4.6	73.5 ± 4.9	44.6 ± 3.0	nil	nil	nil
	1.0	nil	16.5 ± 1.0	89.5 ± 6.0	90.8 ± 6.0	67.2 ± 4.5	nil	nil	nil
[L_2_Co]	0.5	nil	nil	26.8 ± 1.7	36.5 ± 2.4	33.2 ± 2.2	nil	nil	nil
	1.0	nil	nil	69.9 ± 4.7	78.9 ± 5.3	70.1 ± 4.6	nil	nil	nil
BMMB	0.5	nil	nil	nil	nil	nil	nil	nil	nil
	1.0	nil	nil	nil	nil	nil	nil	nil	nil

**Table 7:** Antimicrobial results (zone of inhibition, diameter in cm) of MBOBT and its complexes using gel-diffusion method.

Compound	Bacteria							
	*Staphylococcus aureus*	*Staphylococcus hominis*	*Bacillus* sp^1^	*Bacillus* sp^2^	*Bacillus* sp^3^	*Escherichia coli*	*Salmonella* sp^1^	*Salmonella* sp^2^
[L_2_Z]⋅H_2_O	nil	nil	3 ± 0.1	1.8 ± 0.05	1.5 ± 0.05	nil	1.2 ± 0.04	nil
[L_2_Cu]	1 ± 0.03	1.2 ± 0.04	3.2 ± 0.1	2 ± 0.06	2.1 ± 0.06	1.2 ± 0.04	1.9 ± 0.06	nil
[L_2_Ni]	1.2 ± 0.04	1 ± 0.03	0.9 ± 0.03	0.9 ± 0.03	1 ± 0.04	nil	1.4 ± 0.05	nil
[L_2_Co]	0.9 ± 0.03	1 ± 0.03	1.2 ± 0.04	1.2 ± 0.04	0.9 ± 0.03	0.9 ± 0.03	nil	nil
BMMB	nil	nil	nil	nil	nil	nil	nil	nil
